# Supplementary material for: Causal Relationship between Aging and Anorexia Nervosa: A White-Matter-Microstructure-Mediated Mendelian Randomization Analysis
Source: Biomedicines. 2024 Aug 16;12(8):1874. doi: 10.3390/biomedicines12081874 (PMC11351342; doi:10.3390/biomedicines12081874)
Supplement: Supplementary file 1 [file biomedicines-12-01874-s001.zip › Supplementary Figure/Figure S4/Singe SNP plot of MR analysis for TL to ubm-b-1558.pdf]

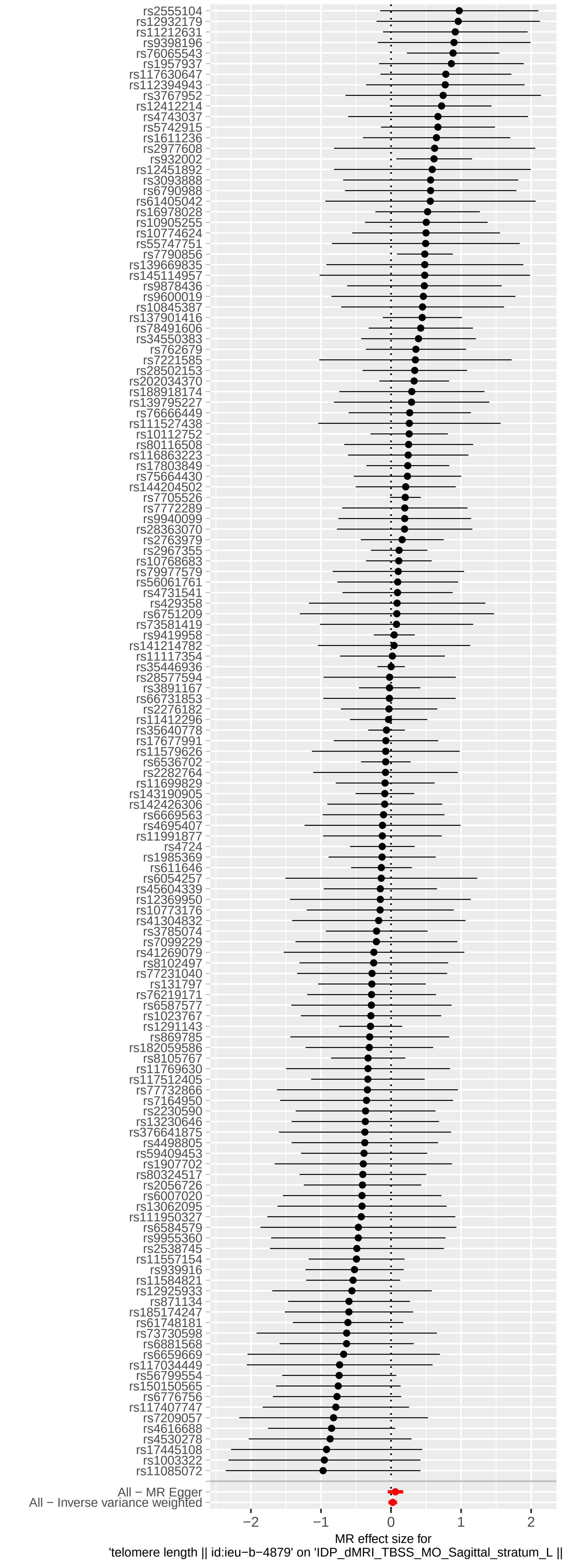
rs2555104
rs12932179
rs11212631
rs9398196
rs76065543
rs1957937
rs117630647
rs112394943
rs3767952
rs12412214
rs4743037
rs5742915
rs1611236
rs2977608
rs932002
rs12451892
rs3093888
rs6790988
rs61405042
rs16978028
rs10905255
rs10774624
rs55747751
rs7790856
rs139669835
rs145114957
rs9878436
rs9600019
rs10845387
rs137901416
rs78491606
rs34550383
rs762679
rs7221585
rs28502153
rs202034370
rs188918174
rs139795227
rs76666449
rs111527438
rs10112752
rs80116508
rs116863223
rs17803849
rs75664430
rs144204502
rs7705526
rs7772289
rs9940099
rs28363070
rs2763979
rs2967355
rs10768683
rs79977579
rs56061761
rs4731541
rs429358
rs6751209
rs73581419
rs9419958
rs141214782
rs11117354
rs35446936
rs28577594
rs3891167
rs66731853
rs2276182
rs11412296
rs35640778
rs17677991
rs11579626
rs6536702
rs2282764
rs11699829
rs143190905
rs142426306
rs6669563
rs4695407
rs11991877
rs4724
rs1985369
rs611646
rs6054257
rs45604339
rs12369950
rs10773176
rs41304832
rs3785074
rs7099229
rs41269079
rs8102497
rs77231040
rs131797
rs76219171
rs6587577
rs1023767
rs1291143
rs869785
rs182059586
rs8105767
rs11769630
rs117512405
rs77732866
rs7164950
rs2230590
rs13230646
rs376641875
rs4498805
rs59409453
rs1907702
rs80324517
rs2056726
rs6007020
rs13062095
rs111950327
rs6584579
rs9955360
rs2538745
rs11557154
rs939916
rs11584821
rs12925933
rs871134
rs185174247
rs61748181
rs73730598
rs6881568
rs6659669
rs117034449
rs56799554
rs150150565
rs6776756
rs117407747
rs7209057
rs4616688
rs4530278
rs17445108
rs1003322
rs11085072
All − MR Egger
All − Inverse variance weighted
−2
−1
0
1
2
MR effect size for
'telomere length || id:ieu−b−4879' on 'IDP_dMRI_TBSS_MO_Sagittal_stratum_L ||'
